# Attenuation Correction Using Template PET Registration for Brain PET: A Proof-of-Concept Study

**DOI:** 10.3390/jimaging9010002

**Published:** 2022-12-21

**Authors:** Markus Jehl, Ekaterina Mikhaylova, Valerie Treyer, Marlena Hofbauer, Martin Hüllner, Philipp A. Kaufmann, Alfred Buck, Geoff Warnock, Viet Dao, Charalampos Tsoumpas, Daniel Deidda, Kris Thielemans, Max Ludwig Ahnen, Jannis Fischer

**Affiliations:** 1 Positrigo AG, 8005 Zurich, Switzerland; 2 Department of Nuclear Medicine, University Hospital Zurich, 8091 Zurich, Switzerland; 3 Institute for Regenerative Medicine, University of Zurich, 8006 Zurich, Switzerland; 4 PMOD Technologies LLC, 8117 Faellanden, Switzerland; 5 Department of Statistics, School of Mathematics, University of Leeds, Leeds LS2 9JT, UK; 6 Department of Nuclear Medicine and Molecular Imaging, University Medical Center Groningen, University of Groningen, 9713 GZ Groningen, The Netherlands; 7 National Physical Laboratory, Teddington TW11 0LW, UK; 8 Institute of Nuclear Medicine, University College London, London NW1 2BU, UK; 9 Centre for Medical Image Computing, UCL, Gower Street, London WC1E 6BT, UK; 10 Algorithms Software Consulting Ltd., London SW15 5HX, UK

**Keywords:** tomography, attenuation correction, image reconstruction, brain, PET, STIR, Nifty-Reg, registration

## Abstract

NeuroLF is a dedicated brain PET system with an octagonal prism shape housed in a scanner head that can be positioned around a patient’s head. Because it does not have MR or CT capabilities, attenuation correction based on an estimation of the attenuation map is a crucial feature. In this article, we demonstrate this method on [^18^F]FDG PET brain scans performed with a low-resolution proof of concept prototype of NeuroLF called BPET. We perform an affine registration of a template PET scan to the uncorrected emission image, and then apply the resulting transform to the corresponding template attenuation map. Using a whole-body PET/CT system as reference, we quantitively show that this method yields comparable image quality (0.893 average correlation to reference scan) to using the reference µ-map as obtained from the CT scan of the imaged patient (0.908 average correlation). We conclude from this initial study that attenuation correction using template registration instead of a patient CT delivers similar results and is an option for patients undergoing brain PET.

## 1. Introduction

Image reconstruction in PET typically requires many correction steps to produce a quantitative image from the measured coincidence pairs. These include hardware-specific corrections such as detector efficiency and geometric normalization, but also random coincidence correction, scatter correction and attenuation correction. On PET/CT systems, the attenuation correction is routinely done by converting CT images to attenuation maps (µ-maps) corresponding to the relevant energy window around 511 keV. PET/MR and PET-only systems need to obtain the µ-maps through other means. The most common approaches [1,2] are to perform transmission scans [3], segmentation methods, atlas-/template-based methods [4,5] and, more recently, methods working directly on uncorrected PET data, such as simultaneous reconstruction of emission and attenuation [6] and deep-learning [2].

NeuroLF is an ultracompact dedicated brain PET system designed to provide comparable image quality to existing full-body PET systems with around 25 cm axial length. It consists of a patient positioning system and a scanning unit to position the scanner head around the patient’s head. The system will make functional brain imaging more affordable for diseases such as dementia, epilepsy, encephalitis, brain cancer and Parkinson. In NeuroLF it would be very attractive to import CT- or MR-based attenuation maps, but generally the attenuation map will have to be computed based on the uncorrected reconstruction of the PET emission data. Of the above-mentioned options, the atlas- or template-based one was considered the most appropriate at this stage, since simultaneous reconstruction techniques such as MLAA [6,7] and deep-learning approaches are technically more complex, and sometimes require TOF which NeuroLF will not initially have. Additionally, atlas- and template-based methods were shown to produce very good results [4,5], especially when using templates instead of atlases. AI approaches will certainly be interesting for NeuroLF in future, since they have already shown very promising results [2] and are improving rapidly due to the active research field.

All methods compared in article [4] used the MR image for atlas or template registration, which has the benefit that they are independent of the radiotracer used for the PET. NeuroLF does not usually have MR data for a scanned patient (only if they have been acquired elsewhere and imported), which means that the template registration should be performed from PET to PET. This article is investigating the feasibility and stability of using a template-based approach for PET-only attenuation correction. We test the proposed method on real patient data acquired with a prototype system (BPET) and different PET/CT template [^18^F]FDG datasets. We show that the proposed method is stable, works with a variety of templates, and that the resulting image quality is equivalent to using CT-based attenuation correction.

## 2. Materials and Methods

### 2.1. Materials

#### 2.1.1. PET Scanner

**Figure 1:**
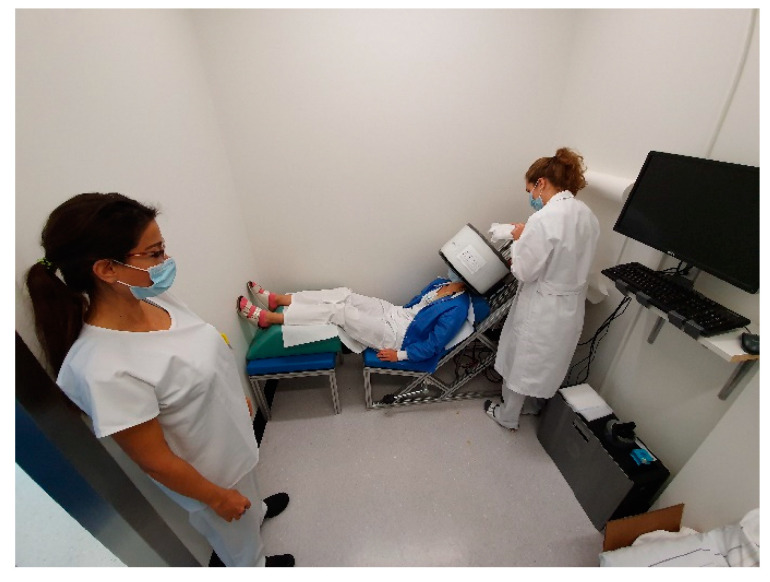
Picture of BPET with three medical technologists practicing its use during the site initiation visit.

Since NeuroLF is still in development, this work on the attenuation correction is performed on data obtained from a proof-of-concept prototype system called BPET (Figure 1), which has a lower spatial and temporal resolution.

BPET has an octagonal prism shape, with each of the eight modules housing 30 (axially) by 24 (transaxially) LYSO crystals with 10 mm length and face dimensions of 4.1 mm × 4.1 mm. These are coupled to silicon photo-multiplier (SiPM) arrays that read-out events using light-sharing, where 3 × 3 SiPMs are interpreting the signal from 6 × 6 crystals to assign measured events to the correct crystal using a light-sharing algorithm. BPET has an axial length of 128 mm and a module to module flat-to-flat diameter of 254 mm [8].

#### 2.1.2. Collected Data

The data used in this article were collected during the first clinical trial of BPET, where eight [^18^F]FDG patients first had their clinically prescribed scan with a whole-body PET/CT system (which constitutes the *reference* PET in this article) and were subsequently scanned with BPET. Four BPET patient scans were taken with light sharing disabled and were excluded from the analysis, the other four will be referred to as patients P1, P2, P3 and P4 from now on and are the BPET datasets used in this study. The reference PET and CT pairs of the four excluded patients were instead used as independent *template* data (named T1, T2, T3 and T4 henceforth). The reference PET images were computed by the PET/CT system (Discovery MI, 6-ring configuration, GE Healthcare, Waukesha, WI, USA) from a 5 min acquisition and included attenuation and scatter correction based on the CT. The acquisition time for BPET was always 15 min, with activity differing for the imaged patients. All data acquisitions were performed at University Hospital Zürich, with ethical approval by KEK Zürich. The clinicaltrials.gov (accessed on 10 December 2022) identifier is NCT04511546 [9].

### 2.2. Methods

#### 2.2.1. Image Reconstruction

Images were reconstructed using the open-source Software for Tomographic Image Reconstruction (STIR) [10], through its Python interface. We used the new scanner geometry modelling feature of STIR called “BlocksOnCylindrical” which was recently developed to reconstruct data obtained by non-cylindrical PET scanners [11,12]. The data were corrected for random events (single rate method [13]), were normalized for geometry (obtained from a scan of a homogeneous cylindrical phantom) and detector efficiencies (computed from singles), and attenuation and scatter correction were performed using the attenuation maps obtained with the method described in this article. The scatter correction was based on the single scatter simulation functionality in STIR, and was scaled up by the average fraction of total scatter events over single scatter events observed in Monte-Carlo simulations in GATE (GEANT4 Application to Tomographic Emission) [14] for the BPET geometry and cylindrical, water-filled phantoms of various diameters around 20 cm (this factor was 1.1).

#### 2.2.2. Attenuation Map Computation

**Figure 2:**
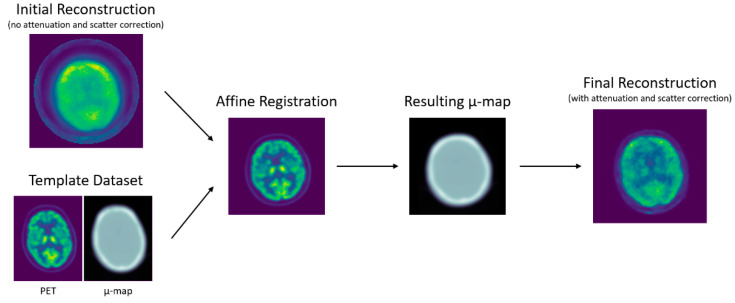
Diagram of the reconstruction workflow, where the template PET and uncorrected BPET image are registered, and the equivalently deformed µ-map is used for attenuation (and scatter) correction. The *initial reconstruction* is the BPET emission image that was reconstructed without attenuation and scatter correction. The *template dataset* is a set of PET image and CT-generated µ-map from the reference PET/CT scanner. The *affine registration* is the template PET after being registered to the initial reconstruction, and the *resulting µ-map* was obtained by applying the same transformation to the template µ-map. This µ-map was then used to perform the attenuation and scatter correction to obtain the *final reconstruction*.

The attenuation map (µ-map) computation consisted of three steps: template preparation, PET-to-PET registration, µ-map deformation (Figure 2). All volumetric image registrations and deformations were performed with the open-source software Nifty-Reg [15,16], which uses a coarse-to-fine block matching to perform rigid and affine registrations. Alternative methods for registration were not investigated yet, but could be of interest [17,18,19].

*Template preparation* only needed to be performed once, and then the templates could be reused for all PET reconstructions. First, the PET and CT scans of the same patient were brought to the same image dimensions using the rigid registration option of the reg_aladin executable in Nifty-Reg. Subsequently, the CT scan was translated to a µ-map with the STIR class HUToMuImageProcessor which uses bi-linear scaling [20]. The intercepts and slopes used for the bi-linear scaling were 0.096 cm^−1^ and 9.6 × 10^−5^ cm^−1^/HU for intensities below 50 Hounsfield Units (HU), and 0.092 cm^−1^ and 5.11 × 10^−5^ above. Finally, the µ-map was blurred with an isotropic 4.5 mm Gaussian 3D kernel to adapt its resolution to the resolution of BPET [21]. For the results in Section 3.1, the reference CT and PET pair for the first of the independently scanned patients was used as the template (T1). To evaluate the stability of the presented attenuation correction method, reference CT and PET pairs of the other three independent FDG datasets were also tested as template data (T2, T3 and T4) in Section 3.2.

During reconstructions, the template data then needed to be adjusted to the patient’s head geometry. Theoretically, this could have been performed in one step by registering the template µ-map directly to the initial BPET reconstruction (without attenuation and scatter correction). However, we found the data in the scalp region of the BPET images was insufficient to provide Nifty-Reg with enough structural information to compute an accurate registration. Therefore, the template [^18^F]FDG-PET image (with attenuation correction) was registered to the initial BPET image first (*PET-to-PET*), using the affine reg_aladin implementation. The difference that the template PET image was attenuation and scatter corrected, while the BPET image was not, did not noticeably impact the registration because these corrections change intensity levels within the image, but not the location of higher-level structural features such as brain lobes, scalp, and sinuses, which have the strongest impact on the registration algorithm.

Finally, the *template µ-map was deformed* by applying the affine transformation obtained in the PET-to-PET registration using the reg_resample executable in Nifty-Reg. This deformation automatically resamples the template µ-map to the coordinate space and dimensions of the BPET reconstructions, which makes it straight-forward to use in the STIR reconstruction pipeline. The PET-to-PET registration and the µ-map deformation generally took around 10 to 20 s, depending on the dimensions of the PET image and the template PET and CT data.

For comparison, we also reconstructed images with the µ-map computed from the CT of the same patients that were scanned with BPET. These were prepared as described in the paragraphs above, with the only difference that all registrations were performed rigidly.

#### 2.2.3. Image Quality Metrics

**Figure 3:**
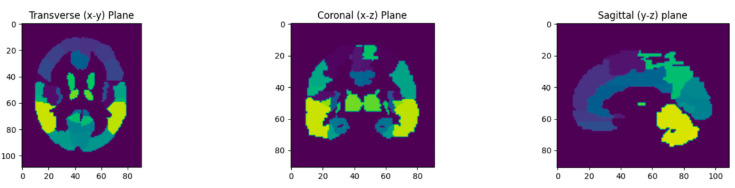
The volumes of interest (VOIs) used for the image quality analysis, as defined in the AAL Merged Atlas in PMOD. Colors indicate discrete VOI numbering from 1 to 71 (with four indices not present).

To assess the quality of the reconstructed BPET images, we compared them to the reference PET scans obtained with the full-body PET/CT system prior to the BPET data acquisition. This was done by comparing the relative voxel intensities in a collection of 67 volumes of interest (VOIs) defined on the AAL Merged Atlas [22,23]. For this, we exported the PET (“SPM5 derived PET template”) and VOI atlas (“AAL Merged Atlas”) images (Figure 3) from PMOD (PMOD Technologies, Zürich, Switzerland), and registered the BPET and the reference PET image to the PMOD PET atlas using the affine implementation of reg_aladin. Then, the PMOD VOI atlas was used to index the VOIs in the BPET and reference PET images to compute the average intensity within each VOI and divide it by the average intensity in the union of all VOIs. The reference PET images were smoothed with an isotropic 4.7 mm Gaussian 3D kernel (pre-registration), to approximately match the resolution of BPET which is between 4 mm and 9 mm, depending on the axial location [8]. The BPET images were filtered with a median filter with a kernel size of 5 × 5 × 5, to reduce salt and pepper noise observed in unfiltered reconstructions.

The resulting 67 relative VOI intensities were then plotted against each other for BPET and reference PET, and a linear regression was computed on the point cloud. A perfect fit would result in a slope of 1 and an intercept of 0.

For the Bland–Altman analysis, the difference in relative VOI intensity (BPET minus reference PET) was divided by their mid-point (labelled “mean VOI activity” on the x-axis). The mean value of this scaled difference was computed and was plotted as a green bar (for a perfect fit it would be 0), and 1.96 times the standard deviation (SD) were plotted either side in orange to show the range within which 95% of the data lie (the closer the lines, the better the fit). This plot is useful to evaluate the variability in the VOI activity accuracy, as well as visualizing whether low or high activity regions are systematically over- or underestimated. Finally, the Pearson correlation coefficient was computed across all relative VOI intensities.

## 3. Results

In the first section, the first template dataset T1 was used for all reconstructions and compared to the patient-specific attenuation map. In the second section, the differences between templates T1–4 are analysed.

### 3.1. Comparison of Patient-Specific Attenuation Map vs. Generic Template Attenuation Map

**Figure 4:**
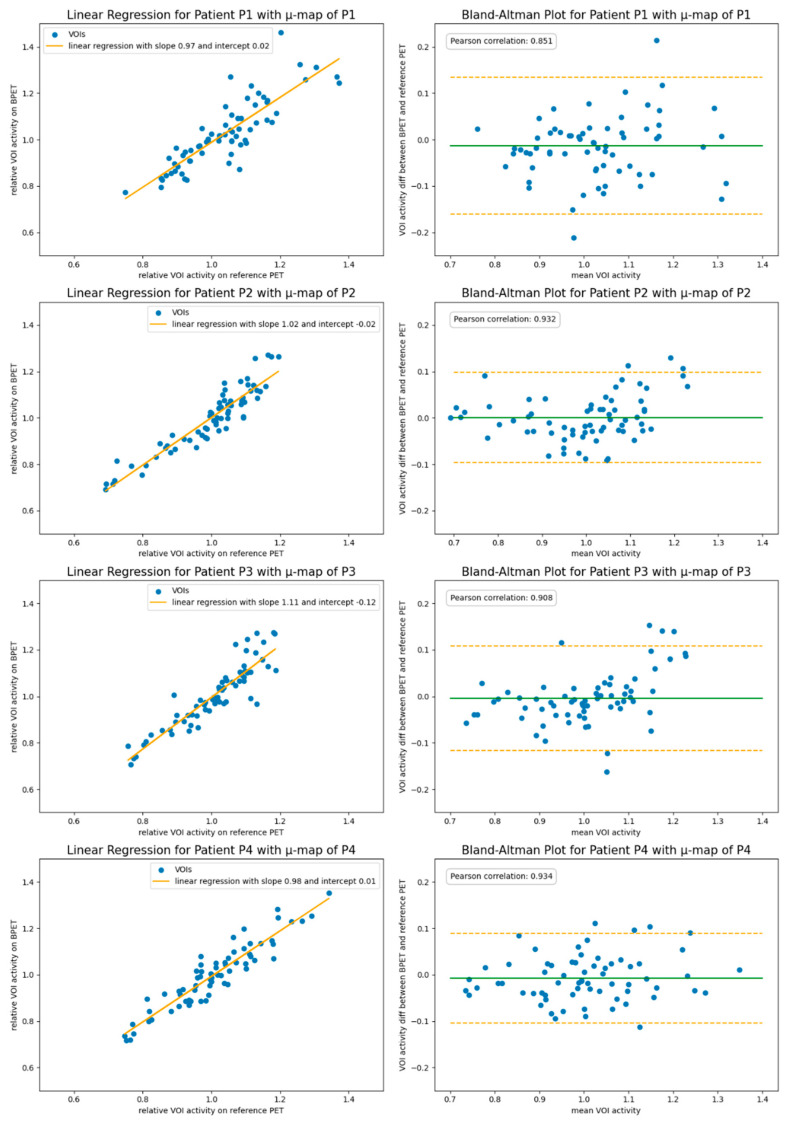
The VOI comparison of reconstructions using the patient-specific reference CT scan for the attenuation correction. Shown left are the linear regression plots. Shown in the right column are Bland–Altman plots indicating the 1.96 SD bars (orange) at either side of the mean intensity ratio (green).

The images reconstructed with the attenuation correction obtained from the patient-specific CT reference scan had an average linear regression slope of 1.01 and an intercept of −0.015 for the VOI comparison, with the reference scans obtained with a clinical GE scanner (Figure 4). The largest mismatch with the reference images was observed for patient P3 with a slope of 1.07 and an intercept of −0.07. The average correlation of the relative VOI values was 0.908.

**Figure 5:**
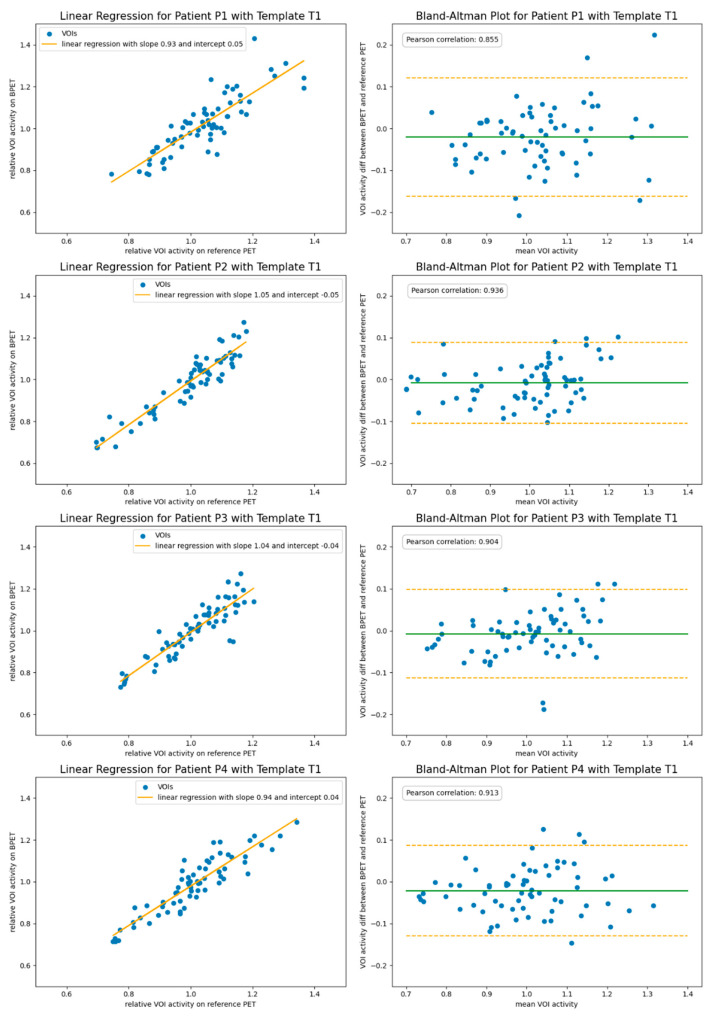
The VOI comparison of reconstructions using the independent template µ-map T1 for the attenuation correction with reconstructions from the reference PET/CT system. In the left column are the linear regression plots. Shown in the right column are Bland–Altman plots indicating the 1.96 SD bars (orange) at either side of the mean intensity ratio (green).

The images reconstructed with the estimated attenuation correction obtained by performing an affine registration of the independent template PET/CT dataset T1 had an average linear regression slope of 0.99 and an intercept of 0.00 for the VOI comparison with the reference scans (Figure 5). The average correlation of the relative VOI values was 0.902.

**Figure 6:**
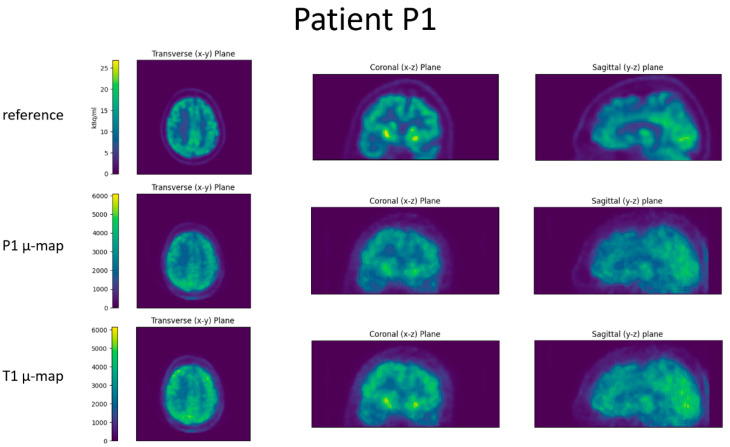
Slices through the reconstructed images for patient P1. Top row is the reference PET scan, middle row the BPET reconstruction using the patient-specific µ-map (from CT of patient P1), bottom row the BPET reconstruction using an independent template µ-map fitted with the method presented in this article.

**Figure 7:**
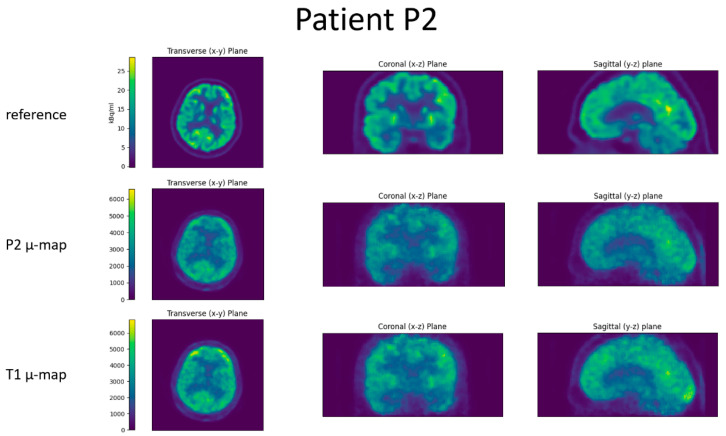
Slices through the reconstructed images for patient P2. Top row is the reference PET scan, middle row the BPET reconstruction using the patient-specific µ-map (from CT of patient P2), bottom row the BPET reconstruction using an independent template µ-map fitted with the method presented in this article.

**Figure 8:**
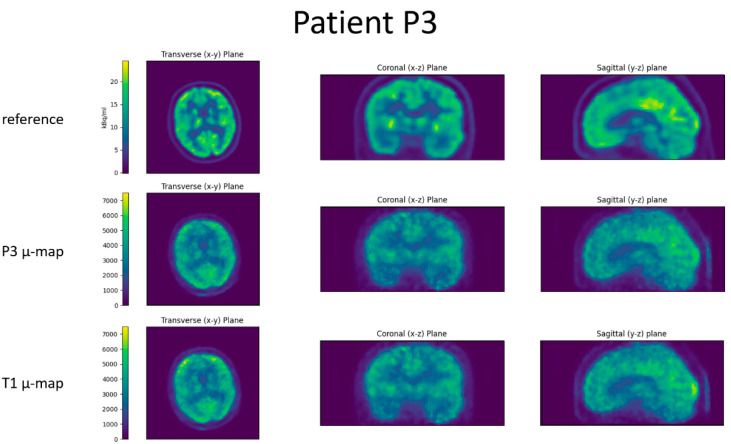
Slices through the reconstructed images for patient P3. Top row is the reference PET scan, middle row the BPET reconstruction using the patient-specific µ-map (from CT of patient P3), bottom row the BPET reconstruction using an independent template µ-map fitted with the method presented in this article.

**Figure 9:**
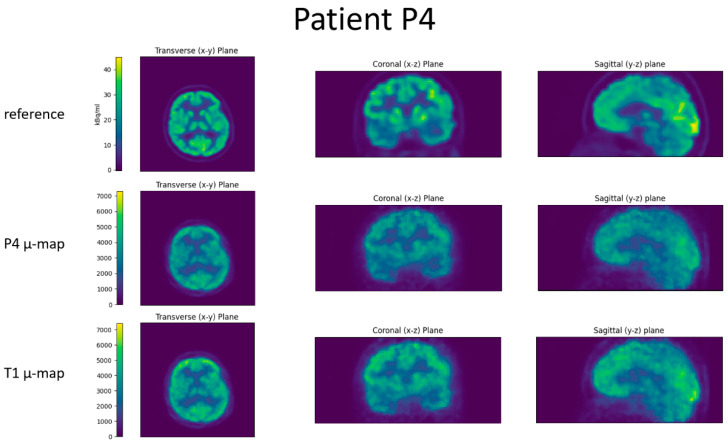
Slices through the reconstructed images for patient P4. Top row is the reference PET scan, middle row the BPET reconstruction using the patient-specific µ-map (from CT of patient P4), bottom row the BPET reconstruction using an independent template µ-map fitted with the method presented in this article.

For all four patients, the reference image, the reconstructed BPET image with the patient-specific µ-map, and the reconstructed BPET image with the µ-map computed from the independent T1 template dataset are shown in Figure 6, Figure 7, Figure 8 and Figure 9. In these figures, the reference images are not smoothed. The BPET images are smoothed by a median filter with kernel size 3 × 3 × 3 to reduce the salt and pepper noise.

### 3.2. Requirements for Template Attenuation Map

**Figure 10:**
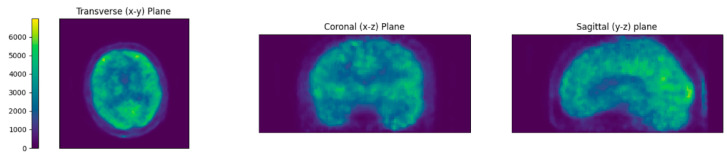
Slices through the reconstructed image for patient P3 with template T2. When compared to the reconstructions in Figure 8, it becomes evident that the slightly lower correlation coefficient 0.8 does not indicate a lower image quality.

For the results in the previous section, the reference CT and PET pair for the first of the independently scanned patients was used as the template (T1). We now compare these to the results with templates T2, T3 and T4. T2 gave similarly good results than T1, while the other two templates gave good results for two patients and worse results for the other two (Table 1). In the following paragraphs we will study these instances with worse image quality metrics in detail to understand the implications on the choice of template, but we would like to note that the images obtained in these cases are still comparable to the ones with the patient-specific µ-map (Figure 10).

**Figure 11:**
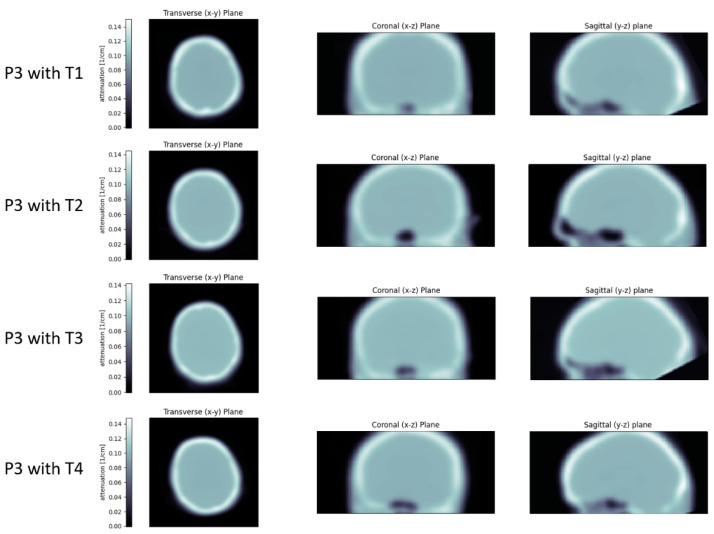
The attenuation maps obtained for patient P3 with the four templates. It is clearly visible that a part of the neck is not covered by the reference PET/CT scan of template T3 (and to a lesser degree also of template T1), and therefore results in a gap in the attenuation map.

The cause for some of these outliers was likely the coverage of the template PET/CT dataset: some reference scans were performed with the head tilted backwards, resulting in a partial loss of the inferior occipital part of the head. When registering these templates to the BPET reconstructions, the resulting attenuation map contains blank areas and is therefore inaccurate (Figure 11). This can be easily remedied by using a template CT with sufficient coverage.

**Figure 12:**
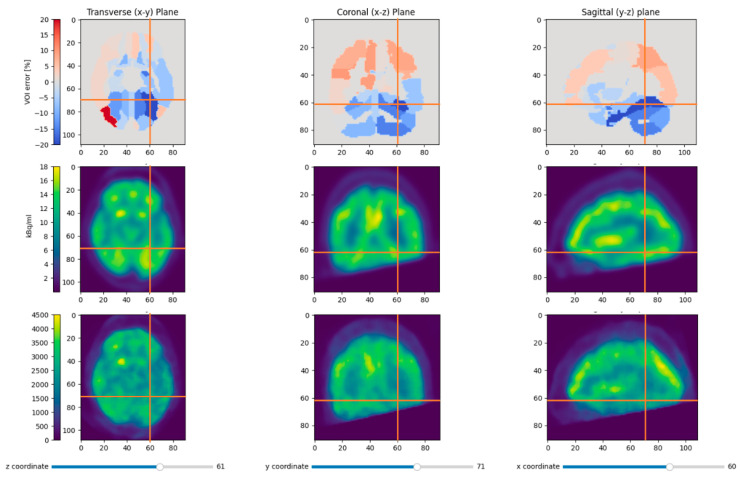
This figure shows the relative VOI deviations for patient P1 with the patient-specific attenuation map in the first row. The middle row shows the smoothed reference PET and the last row the median-filtered BPET reconstruction. Most regions with the largest errors (left cerebellar hemisphere and left fusiform gyrus) were near the area that was outside the BPET field of view due to inaccurate positioning of the patient.

**Figure 13:**
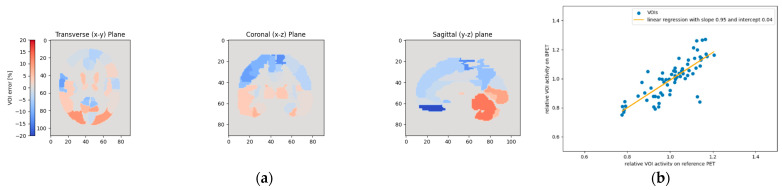
This figure shows the relative VOI deviations when using template T2 for patient P3. By far the highest absolute difference between the BPET image and the reference PET is observed in the lower anterior of the brain (left and right gyrus rectus) (**a**). These two VOIs with the highest deviations can be seen on the linear regression plot (**b**) on the lower right.

**Figure 14:**
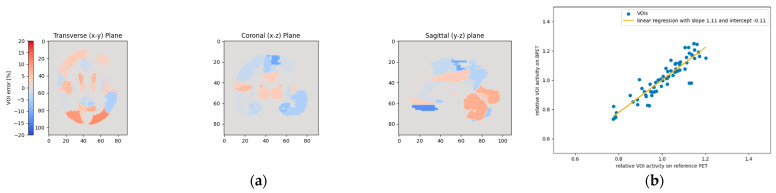
This figure shows the relative VOI deviations when using template T4 for patient P3. As in Figure 13, the left and right gyrus rectus VOIs experienced the largest deviations (**a**). However, these two VOIs with the highest deviations are less pronounced than in Figure 13, as also noticeable on the linear regression plot (**b**).

When looking at the correlation coefficient instead of the linear regression, patient P1 always looks worse than the others, irrespective of the template dataset used (Table 2). This is therefore not explained by the attenuation correction, but hints to another issue, most likely data quality due to the cropping of parts of the cerebellum and a temporal lobe (Figure 12). The only instance where the choice of template had a significant impact on the correlation coefficient was the combination of patient P3 and template T2. To investigate this instance where the correlation was lower, the VOIs were colored according to the intensity difference between reference PET and BPET (Figure 13). There are two regions in the lower front of the brain (left and right gyrus rectus) that experience the largest deviations by a margin. These regions also experience the largest deviations when other templates are used (Figure 14), but the relative difference to other areas is smaller. In this frontal region of the brain, the T2 template had more pronounced paranasal sinuses compared to the other templates (Figure 11), which could explain the larger deviations. Nonetheless, the reconstructed images do not differ noticeably from the ones obtained with another template (Figure 10).

## 4. Discussion

We have presented the use of template PET/CT image pairs for computing an attenuation map during PET image reconstruction. The template PET was registered to the uncorrected image reconstruction of a dedicated brain PET-only scanner, and the same deformation was applied to the template CT to obtain the µ-map. We showed good quality reconstructions for four different patients, and using four different independent template PET/CT pairs, indicating that this technique works reliably for [^18^F]FDG brain PET scans. A couple of specific combinations of patient and template data resulted in a slightly worse linear regression fit or lower correlation coefficient, but the resulting images only looked marginally inferior. To reduce the likelihood of such outliers, the template dataset with the best scores across the total test data can be chosen—since good scores across all test patients can indicate a more general geometry that fits well with most head geometries.

The data used in this paper were obtained from the proof-of-concept prototype BPET. Based on clinical results and clinician’s feedback for BPET, NeuroLF was designed with a larger axial field-of-view, smaller light-sharing group, smaller crystals for higher spatial resolution, an updated readout ASIC, more SiPM readout area for improved light sampling and therefore improved timing resolution, and overall usability improvements. Each of the eight NeuroLF modules will house 48 (axially) by 32 (transaxially) LYSO crystals of configurable length 10 mm, 15 mm or 20 mm, and face dimensions of 3.19 mm × 3.19 mm. These are coupled to SiPM arrays that read-out events using light-sharing, where four SiPMs are used to identify events in a 4 by 4 grid of crystals. NeuroLF will also have a larger field of view with an axial length of 163 mm and module to module flat-to-flat diameter of 268 mm. We are planning to collect more FDG data with NeuroLF, to repeat this analysis on a larger sample size and with better image quality compared to BPET.

Obviously, brain PET scans are not limited to [^18^F]FDG. We intend on repeating this analysis with other radiopharmaceuticals, in particular for [^18^F]-based Aß-tracers, [^18^F]-based tau-tracers, [^18^F]FET, or [^11^C]MET. Initial results with affine registration of PET images of these tracers look promising.

Nifty-Reg also supports a non-linear deformable registration [24], which was tested initially as part of the work presented here. However, this was more time-consuming and produced less reliable results as the more constrained affine registration used here. Furthermore, the good results obtained with the affine registration of four different template datasets indicate that the attenuation map used in brain PET does not need to be extremely accurate, as long as it contains all relevant tissues in the approximate locations. However, we have observed ±10% intensity variations in reconstructions using different templates. These only affect quantification accuracy, but we are working on methods to mitigate this effect.

While the results presented in this article suggest that a single template is sufficient, there is a potential risk of failure for uncommon input data (e.g., [25]). As with any computational attenuation correction method, it is very difficult to make these methods account for all such eventualities, hence it is good practice to compare attenuation corrected reconstructions with unusual activity distributions to reconstructions without attenuation correction. Methods of detecting unusual data automatically will be investigated in the future. Furthermore, it may be worthwhile to compute the quality of registration, to spot potential outliers or to select the best among a group of available templates. This could even be extended to computing local quality measures and fusing multiple templates [26], at the cost of computation time. Alternatively, consistency conditions could be used to select the best template [27].

## Figures and Tables

**Table 1:** Summary of linear regression results for comparison of BPET reconstructions with reference PET. The linear regression is given as a line equation with slope × x + intercept. The “correct µ” column shows the results with the µ-map obtained from the CT of the patient that was imaged.

Patient ID	Correct µ	T1	T2	T3	T4
**P1**	0.97x + 0.02	0.93x + 0.05	1.00x − 0.01	0.98x + 0.01	0.97x + 0.01
**P2**	1.02x − 0.02	1.05x − 0.05	0.94x + 0.06	1.11x − 0.10 ^1^	1.13x − 0.13
**P3**	1.07x − 0.07	1.04x − 0.04	0.95x + 0.04	1.23x − 0.22 ^1^	1.11x − 0.11
**P4**	0.98x + 0.01	0.94x + 0.04	0.96x + 0.03	0.96x + 0.03	0.98x + 0.01

^1^ Accuracy likely impacted by incomplete head coverage of template PET-CT dataset.

**Table 2:** Pearson correlation coefficients between BPET reconstructions and reference PET. The “correct µ” column shows the results with the µ-map obtained from the CT of the patient that was imaged.

Patient ID	Correct µ	T1	T2	T3	T4
**P1**	0.851	0.855	0.881	0.830	0.834
**P2**	0.932	0.936	0.918	0.912	0.928
**P3**	0.916	0.904	0.803	0.885	0.919
**P4**	0.934	0.913	0.919	0.924	0.925

## Data Availability

Contact authors for dataset or code.
